# Safety of Ovarian Tissue Autotransplantation for Cancer Patients

**DOI:** 10.1155/2012/495142

**Published:** 2011-12-27

**Authors:** Laurence Bockstaele, Sophie Tsepelidis, Julie Dechene, Yvon Englert, Isabelle Demeestere

**Affiliations:** ^1^Research Laboratory on Human Reproduction, Faculty of Medicine, Free University of Brussels, 808, Route de Lennik, 1070 Bruxelles, Belgium; ^2^Obstetrics and Gynaecology Department, Erasme Hospital, Route de Lennik, 1070 Bruxelles, Belgium

## Abstract

Cancer treatments can induce premature ovarian failure in almost half of young women suffering from invasive neoplasia. Cryopreservation of ovarian cortex and subsequent autotransplantation of frozen-thawed tissue have emerged as promising alternatives to conventional fertility preservation technologies. However, human ovarian tissue is generally harvested before the administration of gonadotoxic treatment and could be contaminated with malignant cells. The safety of autotransplantation of ovarian cortex remains a major concern for fertility preservation units worldwide. This paper discusses the main tools for detecting disseminated cancer cells currently available, their limitations, and clinical relevance.

## 1. Introduction

Thanks to progress made in the field of cancer treatments, long-term survival of patients has increased considerably over the last decade. Unfortunately, some of these treatments, such as chemo- and radiotherapy, can induce premature ovarian failure. It has been determined that nearly half of women diagnosed with invasive cancer will face premature ovarian failure [1]. Consequently, fertility preservation in reproductive-age women has become a major concern in oncology units during the last decades. However, many patients cannot benefit from classic fertility preservation technologies for medical and/or personal reasons. Cryopreservation of ovarian tissue by slow-freezing followed by autotransplantation of thawed tissue provides an alternative method for fertility preservation in young women and even prepubertal girls [2]. While the exact number of ovarian tissue autotransplantations performed worldwide is unknown, this procedure has resulted in 13 reported births of healthy children [3], including two in our centre [4, 5].

Despite these encouraging results, human ovarian cortex autografts still present some major limitations. A major concern is the possibility of reintroducing malignant cells into the patient. Indeed, as the ovarian biopsy and cryopreservation procedures are ideally performed prior to the administration of chemo- or radiotherapy, there is a risk of ovarian involvement and subsequent retransmission of the disease after autotransplantation. In a Japanese retrospective study performed on autopsy specimens, 22,4% of cancer patients under the age of 40 had ovarian metastases [6]. Most of the metastases affecting ovaries are derived from the gastrointestinal tract, breast cancer, or endometrial cancer [7–9]. It can be argued that these individuals are in advanced stages of the disease compared to women benefiting from ovarian tissue cryopreservation, but this underlines the fact that metastases can be found inside the ovarian tissue of young women. In fact, little is known about the presence of malignant cells inside the graft and the risk of neoplasia retransmission after autotransplantation of cryopreserved ovarian tissue. Of the 13 live births reported in the literature, the autotransplanted frozen-thawed ovarian tissue was derived from 8 cancer patients and two patients treated for benign disease [3]. Among these cancer patients, 4 had Hodgkin's lymphoma (HL), one had breast cancer, one had non-Hodgkin's lymphoma (NHL), one had Ewing sarcoma, and one had neuroectodermic tumour. To date, there are no reports of disease recurrence following the procedure. However, using a mouse lymphoma model, it has been established that lymphoma can be transmitted through the graft even after cryopreservation and thawing of the ovarian tissue if cancer cells are present in the ovary [10]. Lymphoma can be contracted even with one small piece of ovarian tissue (~1 mm^3^) containing cancer cells [10]. The same results have been observed for leukaemia in a rat testis model [11]. In this study, fresh or frozen-thawed testicular cells from leukemic rats were injected in the testis of recipient rats. All of the recipient animals developed signs of leukaemia although a 3–6 day delay was observed in the appearance of symptoms in the frozen-thawed cell transplantation group [11]. Interestingly, it was also demonstrated that only 20 leukemic cells were sufficient to cause leukaemia after 3 weeks in 60% of the animals [11]. These two studies illustrate that malignant cells from haematological cancer can induce relapse in cured patients if these cells are present in the ovarian tissue. Additionally, these results provide evidence that cancer cells are resistant to freezing-thawing process.

The safety of ovarian tissue transplantation in cancer patients should thus be addressed systematically for malignancies with low-to-moderate risk of ovarian implication. Herein, we will discuss the main tools that are currently available for the detection of disseminated cancer cells, specifically classic histology and immunohistochemistry and PCR and xenograft experiments.

## 2. Analysis of Ovarian Tissue by Histology and Immunohistochemistry

In our centre, more than 30% of indications for ovarian tissue cryopreservation concern young patients affected by breast neoplasia. Others have also reported breast cancer as the main indication for fertility preservation [12, 13]. In these cases, the risk of ovarian metastases is considered low to moderate (0.2% to 11%) [14]. Unfortunately, there is no established method for the detection of cancer cells in ovarian tissue. Only a few recent studies have analysed the incidence of ovarian metastasis in breast cancer patients who underwent cryopreservation procedure [15–17]. The authors investigated the presence of breast cancer cells by histology and immunohistochemistry in more than 160 ovarian cortex biopsies originating from 133 women entering the fertility preservation program. One of these studies [17] focused on gross cystic disease fluid protein-15 (GCDFP15) and mammaglobin-1 (MGB-1), two specific markers of breast epithelium that are not normally expressed in ovarian tissue [18–20]. In two additional studies, the authors used broad spectrum cytokeratin (CK) antibodies [15] or CK-7, CK-aecam and markers of ovarian epithelium [16]. In these studies, there was no evidence of malignant infiltration of the ovarian tissue, even in patients with local lymph-node involvement, which were 44% of the cases in the Rosendahl et al. study. The authors prudently concluded that ovarian tissue preservation seems to be a safe procedure in women with early stages of breast cancer, but “*new methods of cancer screening may change their perception of this procedure*” [17]. Similarly, a series of ovarian cortex originating from 28 breast cancer patients benefiting from the fertility preservation program in our centre were analysed by immunohistochemistry. The histology and CK-19 staining revealed no invasion of ovarian tissue by metastatic cells of mammary origin (personal data).

Haematologic malignancies are also reported as a frequent indication for ovarian tissue cryopreservation [21]. In lymphoma cases, the risk of residual disease in the ovary was particularly highlighted by the Shaw et al. study, which reported that ovarian tissue collected from mice with lymphoma could transfer the disease to healthy recipient animals [10]. Many authors have thus addressed the safety of ovarian tissue autotransplantation in lymphoma patients. In addition to ovarian biopsies from patients benefiting from ovarian tissue autotransplantation, ovarian tissues from 79 patients with HL were analysed and demonstrated no histological evidence of malignant contamination [22–25]. The same analysis was performed on the ovarian tissue of NHL patients and did not reveal any ovarian involvement either [22, 24]. Moreover, in xenograft experiments of ovarian tissue from both HL and NHL patients, none of the grafted animals developed the disease [22]. As a consequence of these analyses, the autotransplantation of frozen-thawed ovarian tissue originating from lymphoma patients is currently considered safe [26–29]. In the case of leukaemia, the risk of disease retransmission is much more significant because malignant cells may be present in the patient bloodstream at the time of tissue retrieval. Surprisingly, histological examination of ovarian biopsies from leukaemia patients reveals no invasion of the tissue. In contrast, more sensitive methods of detection have determined that this tissue is, in fact, contaminated by leukaemia cells [21, 24, 30]. This illustrates the limitations of histology and immunohistochemistry in terms of sensitivity of detection. Moreover, these methodologies only examine a small part of the tissue, and, therefore, are necessary but not sufficient to establish the safety of ovarian autotransplantation in cancer patients. As a result of these limitations, complementary approaches to improve the detection of metastatic disease in ovarian tissue are being developed. As discussed below, polymerase chain reaction (PCR) offers several advantages in this context when used in state of the art.

## 3. Molecular Analysis of Ovarian Tissue

Numerous studies detailed above were carried out using only classic histology and immunohistochemistry. However, several recent studies used a complementary molecular approach to improve the sensitivity of detection for disseminated cancer cells. Indeed, quantitative reverse transcription-polymerase chain reaction (RT-PCR) has a high sensitivity and specificity of disseminated cancer cell detection, one cancer cell in up to 10^7^ normal cells, and can be applied to virtually all types of cancer if adequate tissue or cancer-specific molecular markers are available. In clonal diseases, such as leukaemia and NHLs, molecular markers for assessing minimal residual disease can often be identified. In general, these markers are an immunoglobulin gene rearrangement in B-cell lymphoma, a T-cell receptor gene rearrangement in T-cell lymphoma, the BCR-ABL (breakpoint cluster region-Abelson) translocation in chronic myeloid leukaemia (CML), or translocations and mutations in acute lymphoblastic leukaemia (ALL). For these diseases, markers are often, but not always, available, and this approach has been used by several teams on ovarian tissue for the detection of leukaemia and lymphoma cell contamination [21, 24, 30]. In the Dolmans et al. study, no malignant cells were detected by histology in the ovarian tissue of six patients with CML and 12 patients with ALL, whereas ovarian tissue in 33% of CML patients and 70% of ALL patients were found to be positive by quantitative RT-PCR [21]. Moreover, xenograft experiments showed leukemic invasion of grafts originating from 5/12 ALL patients [21]. The same conclusions were made in another study in which histology and multimarker immunohistochemical analyses were both negative for the presence of malignant cells, whereas disease-specific genetic markers were detected by quantitative RT-PCR in 6 of 8 patients with CML or ALL [30]. These results demonstrate the presence of residual disease in a high percentage of the ovarian biopsies from patients with leukaemia, which suggests that autotransplantation of frozen-thawed ovarian tissue in these patients is not safe.

Surprisingly, although breast cancer constitutes the major indication for ovarian tissue cryopreservation in several centres, no molecular analyses of the presence of disseminated breast cancer cells in ovarian tissue have been reported to date. As the safety of ovarian autotransplantation in breast cancer women remains a major concern, we have initiated the validation of quantitative RT-PCR markers specific to breast epithelium using the same method employed for the detection of metastatic cells in the sentinel node. We attempted to evaluate ovarian tissue contamination by metastases of mammary origin and the presence of circulating mammary tumour cells (CTCs) in the peripheral blood of patients retrieved at the time of the cryopreservation procedure. Indeed, the presence and number of CTCs are a poor prognostic factor in terms of relapse, survival, and the presence of micrometastases in several solid neoplasias including breast cancer [31]. Several molecular markers permitting the detection of mammary CTCs by RT-PCR have been described in the literature [32, 33], and we have evaluated the sensitivity and specificity of five of them for the detection of CTCs in peripheral blood and disseminated tumour cells (DTCs) in ovarian tissue. Unfortunately, while the majority of these markers are useful for the detection CTCs, none of them are useful for the detection of mammary micrometastases inside the ovarian cortex due to their strong basal expression in normal tissue (personal data). Additional investigations are necessary to evaluate whether these positive signals are the result of illegitimate transcription or normal expression in the ovarian epithelium. To identify new mammary molecular markers that could be used in ovarian tissue, it would be interesting to explore a group of small noncoding ribonucleic acids (RNAs), called micro-RNA (mi-RNAs). Indeed, some mi-RNAs are expressed in a tissue-specific manner and can be differentially expressed between tumours and normal tissues [34]. These characteristics could be very useful for the detection of disseminated breast cancer cells and also in other neoplasias.

Despite the sensitivity and the fact that this molecular tool can theoretically be applied to all malignancies, quantitative RT-PCR detection of disseminated cancer cells inside ovarian tissue is not devoid of limitations. Indeed, extracting RNA from this dense and fibrous tissue is challenging and, the results obtained from this analysis are highly dependent on the quality of RNA extracted and the efficiency of cDNA synthesis. We have also noticed that genetic markers are not always available for all patients, particularly for ALL patients. In addition, illegitimate transcription has been well described and could lead to false positives. Finally, the clinical relevance of a positive signal inside ovarian tissue has not been established yet. Therefore, further studies are required to evaluate if cancer cells detected in thawed ovarian tissue are viable and the transplantability threshold of these cells. As discussed below, xenograft experiments can partially address these last issues.

In conclusion, it is now clear that the detection sensitivity of disseminated cancer cells can be increased by the use of molecular detection tools, such as quantitative RT-PCR. However, this technique should not be used alone in this context, but only in combination with other detection tools, like immunohistochemistry and/or xenografting.

## 4. Xenotransplantation, a Tool to Evaluate the Safety of Ovarian Tissue Autotransplantation in Cancer Patients

The first clinical attempts at xenotransplantation date back to the 17th century when blood from animals was used to transfuse humans in France and England [35]. In the 19th century, tissues (mainly the skin) and during the 20th century, vascularised organs were attempted to be grafted into humans without success [35]. The reason for these failures was the acute rejection of the transplanted tissue by the immune system [36]. The development of transgenic immunotolerant animal models allowed xenotransplantation to have new insights in the research field. Today, some mice strains have mutations that make them sufficiently immunodeficient to permit xenotransplantation [36]. Among them, nude mice that are athymic, and thus T-cell deficient, [37] and severe combined immunodeficient mice (SCID) carrying an autosomal recessive mutation that severely affect lymphopoiesis, which makes mice that are homozygous for this mutation deficient in B and T lymphocytes [38], are the most frequently used for ovarian tissue xenografts. Many sites have been used for ovarian cortex xenografts, including subcutaneous sites, the bursal cavity, under the kidney capsule, or in the muscle [39–47].

As a risk of reintroducing cancer into remission patients is theoretically possible following autotransplantation of ovarian tissue, xenotransplantation of frozen-thawed ovarian cortical tissue to immunodeficient animal hosts has been suggested as an alternative to assess the safety of the procedure. Xenotransplantation models have initially focussed on studying ovarian follicular development, in which primordial follicles are activated in an immunocompromised animal model and, after initial growth, are transferred to an in vitro culture system [36]. This approach eliminates the risk of cancer cell reintroduction, and, additionally, the problematic unaccomplished phase of primordial follicle culture is bypassed [36]. Unfortunately, the use of xenotransplantation to mature follicles “in vivo” is still not ready to be used in clinical applications, as its safety and ethical issues have yet to be discussed [48].

The use of xenotransplantation to evaluate the risk of reintroducing malignant cells is quite recent [22]. The first in vivo evaluation of residual disease using xenograft models was studied in HL and NHL [22]. No clinical sign of the disease and no microscopic evidence of residual disease were found in animals xenografted with ovarian tissue from patients diagnosed with HL or NHL [22]. Although these results are quite reassuring, they cannot be interpreted as absolute evidence of safety. Some years later, ovarian tissue xenotransplantation to immunodeficient mice was used to evaluate the risk of reintroducing leukaemia, in parallel with histology and quantitative RT-PCR [21]. In fact, as leukaemia is considered as a systemic cancer, malignant cells may be present in the bloodstream and can thus easily migrate to the ovary. Furthermore, a retrospective analysis of an autopsy study demonstrated leukemic invasion of the ovaries in 8,4% of patients [6]. After long-term xenografting (6 months) of frozen-thawed ovarian tissue from patients with CML and ALL into SCID mice, one third of the mice grafted with tissue from ALL patients showed massive macroscopic peritoneal invasion [21]. No malignant cells were microscopically identified in grafts retrieved from mice transplanted with ovarian tissue from CML patients; however, obvious invasion of lymphoblasts was observed in 5 of the 12 mice grafted with ovarian tissue from ALL patients [21]. These results are quite alarming considering all ovarian tissues that were determined be healthy and disease-free following histological analysis preceding the xenograft. Moreover, only histological, and eventually immunohistochemical, analysis is routinely performed prior to autotransplantation of ovarian tissue in a cured patient.

More recently, the safety of ovarian tissue autotransplantation from patients with ovarian tumours was investigated using xenografting to SCID mice [49]. After 24 weeks, no sign of malignancy was detected either macroscopically or histologically [49]. Despite these reassuring results, the risk of reintroducing malignant cells in cases of ovarian cancer is considered high, as bilateral carcinoma is found in approximately 25% of all ovarian cancers [50].

Regarding breast cancer, no study using a xenograft model has been published to date. The first evaluation by histology and immunohistochemical analysis showed no sign of metastases [16, 17]; however, further investigations, using xenograft models, for instance, are still required to confirm the safety of the procedure in breast cancer patients.

## 5. Conclusion

It is now clear that cryopreserved ovarian tissue may harbour malignant cells that could provoke relapse following grafting. Currently available data suggest that autotransplantation of frozen-thawed ovarian tissue is a safe procedure for patients with Hodgkin's disease at the time of ovarian biopsy [26–29]. Indeed, there is no evidence of ovarian involvement in the ovarian cortex of patients undergoing cryopreservation. Moreover, no relapse has been reported in patients after ovarian autotransplantation and spreading to the ovary has only been described in extremely rare cases [51]. However, even though these results are reassuring, it does not mean that there are no risks associated with autotransplantations. Moreover, the safety of the procedure has not yet been established for all other malignancies. If it is clear that autotransplantation of ovarian tissue cannot be proposed for leukemic patients, precautionary decisions have to be taken for all other patients [21, 24, 30]. In the case of other neoplasias, and in particular for breast cancer patients, additional strategies must be developed to determine the safety of this procedure. This implies the identification of (breast) cancer molecular markers that are usable in ovarian tissue and xenograft experiments. We believe that quantitative RT-PCR, despite its limitations, is a promising tool for the detection of micrometastases inside ovarian tissue. Additionally, xenotransplantation studies in immunotolerant mice provide additional information concerning putative ovarian involvement.

However, these tools are not perfect for the detection of micrometastases as they also have some disadvantages. A major limitation of RT-PCR is the interpretation of positive results. Indeed, it has been shown that BCR-ABL mRNA can be detected at very low levels in healthy patients [52, 53]. Similarly, a recent study showed that leukemic markers can be detected in ovarian tissue from ALL patients; however, some mice xenografted with this tissue did not develop neoplasia [21]. As expected, none of the mice grafted with PCR-negative tissue developed leukemic progression either [21]. A potential limitation of xenotransplantation experiments is that a lack of tumour growth in recipient mice could be explained simply by the absence of malignant cells in the very small piece, usually 1 mm^3^ or less, of ovarian cortex typically used for the graft, whereas malignant cells could be present in the rest of the cryopreserved tissue. Indeed, the distribution of malignant cells in the tissue is not homogeneous or uniform. It is therefore of major importance to standardise detection techniques of residual malignant cells and to assess their clinical relevance. To accomplish this, it would be interesting to develop a multicentric and multidisciplinary approach combining molecular and xenograft analyses for pathologies with low-to-moderate risk of ovarian invasion. For cancer patients who cannot benefit from ovarian tissue autotransplantation due to the risk of disease retransmission, alternatives such as in vitro follicle culture [54] and isolated follicles transplantation [55] are promising approaches. However, these alternatives are still in the early stages, and huge research efforts need to be conducted prior to clinical implementation.

 In conclusion, the decision to graft a patient or not must involve a multidisciplinary discussion involving oncologists, gynaecologists, anatomopathologists, and molecular biologists. It is essential to balance the risks and benefits for each patient and remain extremely cautious regarding ovarian cortex autotransplantation. We should keep in mind that although recovering fertility is very important to some patients, reimplantation of contaminated ovarian tissue could be a life-threatening event.
